# Tait’s Operation in Texas

**Published:** 1886-10

**Authors:** 


### TAIT'S OPERATION IN TEXAS.

OUR "esteemed" of the *Courier Record*, has been to Galveston. They say "go away from home to hear the news." He heard some "news" (to us) there. In giving an account of a meeting of the Galveston Medical Club, which he attended, the editor says:

"The President, [Dr. C. H. Wilkinson] stated that Tait's operation has been performed in Texas four times, namely: Once by Dr. ——, of Lampasas; once by Dr. Thompson, of Sherman; once by himself, and now by Dr. Fly, of Galveston. To this list we will add a fifth operation, by Dr. Allen, of Dallas."—*Courier Record*, *Sept. 26th.*

This is certainly a very poor showing for Texas; and, believing that it is incomplete; that the operation has been performed *more than five times in San Antonio alone*, we addressed a note to Dr. Cupples—the Nestor of surgery in Texas—asking for data. To this the doctor very promptly sent the following:

"*Case 1*—By G. G. Watts; menstrual epilepsy; single; aged 23; Taits' operation; death on 5th day; peritonitis. Date, November, 1883. First operation in Texas.

"*Case 2*—By G. Cupples; widow, aged 49, of many years standing; Tait's operation; recovery, with cure of ailments.

"*Case 3*—By Cupples; married woman, aged 22; three years standing; amenorrhœa of ten months; diagnosed adhesions with probable pyo. salpinx, laparotomy; removal of adhesions. Ovaries not seen; very rapid and permanent cure. Painless menstruation on twenty-seventh day after operation.

"*Case 4*—By Cupples; married woman, aged 22; excessive vaginismus; intense dysmenorrhœa from the age of 13; morphia habit. Tait's operation; sutures removed on the fourth day. Complete cure.

"*Case 5*—By F. Herff; married woman about 30 years of age; long standing dysmenorrhœa of agonizing severity; Batty's operation; suppurative peritonitis; womb reopened. irrigation; drainage. Final recovery and cure.

"(Apropos: Dr. Herff made a successful nephrectomy in the person of a young married woman, whom I examined at his request. This was nearly two years ago.)

"Make what use you please of the foregoing, the correctness of which I vouch for, as I operated or assisted in all these cases."

To the above must be added:

By Dr. B. E. Hadra, at Austin. Mrs. M. ——, Tait's operation, recovery; Mrs. G., Tait's operation, died of exhaustion; Mrs. ——, Tait's operation, recovery.

"Three straight Tait's" the doctor calls them.

By Dr. G. W. Christian, Burnet, one Tait's operation.

Including the above, Dr. Hadra has performed laparotomy eighteen times for various purposes.

The above is as far as heard from. We venture Tait's operation has been performed in Texas nearer fifty, than five times. A number of distinguished operators are to be heard from yet; and since our friend, Brooks, has started the subject and put his foot in it, we suggest to him that it would be a good idea—a service to the profession—of interest to his readers, and simple justice to Texas and her surgeons, a field in which he can earn new and additional laurels, to get up statistics on the subject, and we *are sure* he can make a better showing for Texas.

